# CdS-Decorated Porous Anodic SnO_x_ Photoanodes with Enhanced Performance under Visible Light

**DOI:** 10.3390/ma15113848

**Published:** 2022-05-27

**Authors:** Karolina Gawlak, Dominika Popiołek, Marcin Pisarek, Grzegorz D. Sulka, Leszek Zaraska

**Affiliations:** 1 Department of Physical Chemistry and Electrochemistry, Faculty of Chemistry, Jagiellonian University, Gronostajowa 2, 30-387 Krakow, Poland; triksu70@gmail.com (D.P.); sulka@chemia.uj.edu.pl (G.D.S.); 2 Laboratory of Surface Analysis, Institute of Physical Chemistry, Polish Academy of Sciences, Kasprzaka 44/52, 01-224 Warsaw, Poland; mpisarek@ichf.edu.pl

**Keywords:** tin oxides, anodization, porous films, CdS, photoelectrochemistry

## Abstract

Electrochemically generated nanoporous tin oxide films have already been studied as photoanodes in photoelectrochemical water splitting systems. However, up to now, the most significant drawback of such materials was their relatively wide band gap (ca. 3.0 eV), which limits their effective performance in the UV light range. Therefore, here, we present for the first time an effective strategy for sensitization of porous anodic SnO_x_ films with another narrow band gap semiconductor. Nanoporous tin oxide layers were obtained by simple one-step anodic oxidation of metallic Sn in 1 M NaOH followed by further surface decoration with CdS by the successive ionic layer adsorption and reaction (SILAR) method. It was found that the nanoporous morphology of as-anodized SnO_x_ is still preserved after CdS deposition. Such SnO_x_/CdS photoanodes exhibited enhanced photoelectrochemical activity in the visible range compared to unmodified SnO_x_. However, the thermal treatment at 200 °C before the SILAR process was found to be a key factor responsible for the optimal photoresponse of the material.

## 1. Introduction

A significant challenge for the modern world is obtaining clean energy. In this context, the production of hydrogen via photoelectrochemical (PEC) water splitting seems to be extremely promising. The most common PEC systems consist of a semiconducting photoanode being able to absorb light of specific energy, which generates electron–hole pairs. Electrons are then transferred through an external circuit to the cathode (e.g., Pt electrode), where hydrogen evolution occurs. At the same time, photoexcited holes are accumulated on the surface of the photoanode (semiconductor), resulting in water oxidation to gaseous oxygen [1,2,3].

Many scientific efforts are focused on designing electrode materials that fulfill the most criteria for efficient water splitting. One of them is the use of self-supported nanostructured photoanodes formed directly by anodic oxidation (anodization) of particular metals, such as titanium [4,5], zinc [6,7], tungsten [8,9], iron [10,11], or tin [12,13,14].

For photoanodes based on anodically generated nanoporous tin oxide films, numerous challenges have to be overcome, from choosing appropriate electrosynthesis conditions for obtaining continuous nanostructures [15] to non-stoichiometric compositions of this kind of film [12,14]. Different strategies, including controlled thermal treatment [12,14], crystallization in water [16,17], and decoration with other semiconductors [18,19,20], have already been proposed to optimize the photoelectrochemical performance of tin oxide films. Among them, the latter approach, based on the deposition of different narrow band gap semiconductors on the tin oxide nanostructures, is considered especially promising since it results in enhanced absorption of the visible light and facilitates the charge carriers’ separation due to the favorable band alignment. In this context, cadmium sulfide (CdS) seems to be attractive due to a narrow band gap (ca. 2.42 eV) as well as conduction and valence band edges located at more negative potentials compared to SnO_2_ [21,22,23] that could facilitate the transport of holes and electrons to the photoanode surface and current collector, respectively. Several physical and chemical methods have already been proposed for the deposition of CdS thin films [24,25]. Some of them require the use of specified and complex apparatus (e.g., vacuum techniques), while in some other cases, the process is carried out at high temperatures. Bearing in mind that deposition of another semiconductor directly on the anodic tin oxide films is still a challenge due to the presence of metallic Sn support having a relatively low melting point (ca. 230 °C), the ambient temperature methods such as successive ionic layer adsorption and reaction (SILAR) seem to be especially promising [22]. In recent years, several examples of the use of this method for the sensitization of SnO_2_-based photoanodes with CdS have been published [22,26,27]. For instance, Zhang et al. [22] successfully prepared the unique screw-like SnO_2_ nanostructures decorated with CdS, which exhibit the excellent efficiency of photoelectrochemical water splitting under solar radiation. However, according to our best knowledge, the modification of anodically formed nanoporous SnO_x_ films by CdS has not been reported so far.

Therefore, herein we propose for the very first time an easy way to modify the anodic nanoporous SnO_x_ films by deposition of the thin layer of CdS via the SILAR method to improve the photoelectrochemical performance of electrochemically generated tin oxide layers in the visible range.

## 2. Materials and Methods

### 2.1. Synthesis of Nanoporous SnO_x_ Layers

Nanoporous tin oxide (SnO_x_) layers were synthesized via the previously optimized procedure (see ref. [15]). Briefly, Sn foil (98.8%, Goodfellow, Huntingdon, UK, 0.5 mm thick) was cut into specimens with dimensions of ca. 2.5 × 0.5 cm, degreased in ethanol and acetone, and dried. The obtained samples were then subjected to anodic oxidation in a homemade Teflon^TM^ cell. The process was performed in a two-electrode system with Sn sample and Pt grid serving as an anode and cathode, respectively (for a detailed description of the experimental setup, see ref. [28]). Anodization was carried out at room temperature in a 1 M NaOH solution under the constant potential difference of 4 V for 50 min. No electrolyte stirring was applied. After anodization, every sample was carefully washed in deionized water and ethanol and dried in a stream of warm air. Part of the samples was then annealed in air at 200 °C (heating rate of 2 °C min^−1^) in a muffle furnace (FCF 5SHM Z, Czylok, Jastrzębie-Zdrój, Poland) for 2 h. Such annealing conditions were earlier found as those providing the optimal photoelectrochemical performance of anodic SnO_x_ films grown at the aforementioned conditions (see ref. [14]). Both unannealed and annealed samples were taken for further modifications.

### 2.2. Deposition of Cadmium Sulfide

Cadmium sulfide (CdS) was deposited on the surface of tin oxide film by the SILAR method. Before the process, the surface of Sn metal not covered with the anodic oxide film was insulated with paraffin to ensure that only the SnO_x_ layer would be exposed to the solutions with Cd^2+^ and S^2−^ ions. The whole SILAR cycle consisted of four following stages. At first, the specimen was immersed in a 0.05 M Cd(NO_3_)_2_ solution for 4 min. Next, the sample was washed in distilled water for 1 min, moved to a 0.05 M solution of Na_2_S for another 4 min, and again washed in water for 1 min. The whole cycle was repeated four times. After the SILAR procedure, part of the samples was annealed in air at 200 °C with a heating rate of 2 °C min^−1^ for 2 h. Considering the low melting point of the remaining Sn substrate (ca. 230 °C), as well as the noticeable worsening of the photoelectrochemical activity of the unmodified anodic SnO_x_ after annealing in air at 400 °C (for details, see ref. [14]) the same annealing conditions such as after anodization (i.e., 200 °C, 2 h) were applied after CdS deposition. Therefore, four different types of SnO_x_/CdS samples were taken for further examinations, and the labels of particular samples are collected in Table 1. Please note that “-” in the sample’s name refers to lack of annealing, and “a” means that the sample was subjected to the thermal treatment. Therefore, “-/a” indicates the sample that was unannealed before CdS deposition but annealed after modification, etc. Porous anodic SnO_x_ films obtained at the same conditions but unmodified with CdS were also characterized as reference materials.

### 2.3. Characterization of the Obtained Materials

The morphology and composition of as obtained oxide films were examined by using a Field-Emission Scanning Electron Microscope equipped with an Energy-Dispersive Spectroscopy (EDS) system (FE-SEM/EDS, Hitachi S-4700 with a Noran System 7, Tokyo, Japan). X-ray diffraction (XRD) measurements were performed using a Rigaku (Tokyo, Japan) Mini Flex II X-ray diffractometer with monochromatic Cu Kα radiation (λ = 1.5418 Å) in the 2θ range of 20–70° with a scan rate of 3° min^−1^ and a step size of 0.02°. The chemical composition of the surface was verified by X-ray photoelectron spectroscopy (XPS) using an ESCALAB 250 Xi spectrometer (Thermo Scientific, Waltham, MA, USA) with a monochromatic Al_kα_ source (spot size 650 µm). Avantage software (5.9911 ver. Fisher Scientific, Waltham, MA, USA) was used for deconvolution of the measured XPS signals, where the following data processing parameters were applied: a smart function background subtraction (signal intensity), an asymmetric Gaussian/Lorentzian mixed function (fitting procedure), the binding energy (BE) of all measured spectra were corrected in relation to the C1s 285.0 eV carbon peak. UV-Vis diffuse reflectance spectra (DRS) were recorded in the range of 250–800 nm using a Lambda 750S spectrophotometer (Perkin-Elmer, Waltham, MA, USA) equipped with an integrating sphere module. The obtained spectra were then converted to Kubelka–Munk function (F(R)) using PerkinElmer UV WinLab Data Processor and Viewer.

### 2.4. Photoelectrochemical Measurements

The photoelectrochemical activity of the obtained samples was measured using a photoelectric spectrometer equipped with a 150 W Xenon arc lamp combined with a potentiostat (Instytut Fotonowy, Kraków, Poland). The measurements were performed in a Teflon^TM^ cuvette with a quartz window in a typical three-electrode configuration with the obtained semiconducting samples, Pt wire, and a saturated calomel electrode (SCE) serving as working, counter, and reference electrodes, respectively. A borate buffer solution with a pH = 7.4 was used as an electrolyte, and the SCE electrode was introduced to the solution through the Haber–Luggin capillary filled with 1 M KNO_3_. The photocurrent spectra were recorded during sequential illumination of the working electrode with a monochromatic light in the range of 200–550 nm (with a step size of 10 nm) at the potential of 1.0 V vs. SCE.

## 3. Results and Discussion

**Figure 1:**
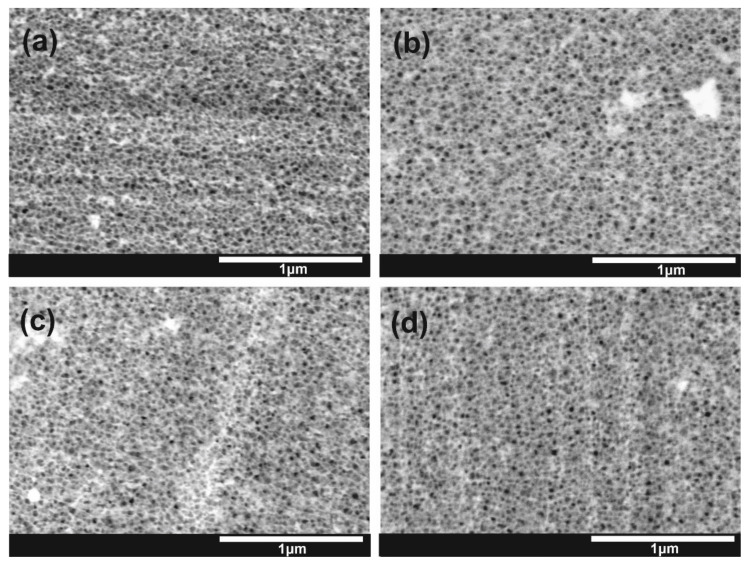
FE-SEM images of different types of SnO_x_ layers after deposition of CdS: -/- (**a**), -/a (**b**), a/- (**c**), and a/a (**d**).

FE-SEM images of all studied types of nanoporous SnO_x_/CdS films (see Table 1) are collected in Figure 1. In every case, the porous morphology of the layer is clearly visible, indicating that the SILAR procedure did not cause significant damage to the nanoporous nature of the as-anodized films (compared with the SEM image of the unmodified SnO_x_ layer presented in Figure S1 in Supplementary Information or with those shown in our previous works [14,15]). The only difference is the presence of some brighter areas, suggesting that some other material could be successfully deposited on the surface of SnO_x_ films. Moreover, the edges of channels are less sharp, and some pores seem to be partially filled with other phases (see higher magnification images shown in Figure S2 in Supplementary Information). However, no significant differences in morphological features of all four types of samples can be seen, which indicates an insignificant effect of the thermal treatment on the morphology of the anodic films (as already proved in our previous paper [14]).

**Figure 2:**
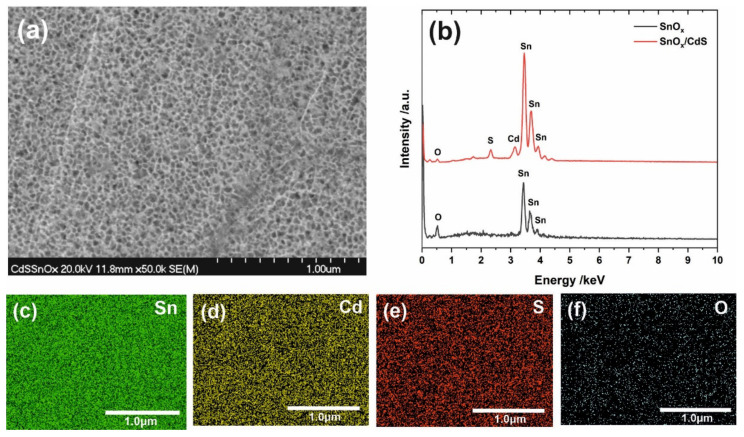
FE-SEM image of annealed nanoporous SnO_x_/CdS **(a)** together with EDS elemental mapping of Sn (**c**), Cd (**d**), S (**e**), and O (**f**). (**b**) shows EDS spectra recorded for anodic SnO_x_ (black line) and SnO_x_/CdS (red line).

EDS spectra of nanoporous SnO_x_ before and after CdS deposition (after annealing) are shown in Figure 2b. Compared to bare SnO_x_, slight peaks at ca. 2.33 eV and 3.15 eV could be observed in the spectrum recorded for SnO_x_/CdS, which can be attributed to S and Cd, respectively. Moreover, elemental EDS mapping (see Figure 2c–e) proved a uniform distribution of Sn, Cd, and S on the surface of the anodic film.

XRD patterns of all studied samples did not confirm the presence of the crystalline cadmium sulfide phase even after annealing at 200 °C in air. Figure S3 in Supplementary Information shows the XRD pattern for the a/a sample with no other maxima except those attributed to metallic Sn and SnO phases. It is caused by the overlapping of CdS maxima with those from tin oxides and metallic Sn and, mostly, the low amount of CdS compared to other phases. Therefore, a detailed composition of the surface of nanoporous SnO_x_/CdS layers was studied by XPS.

**Figure 3:**
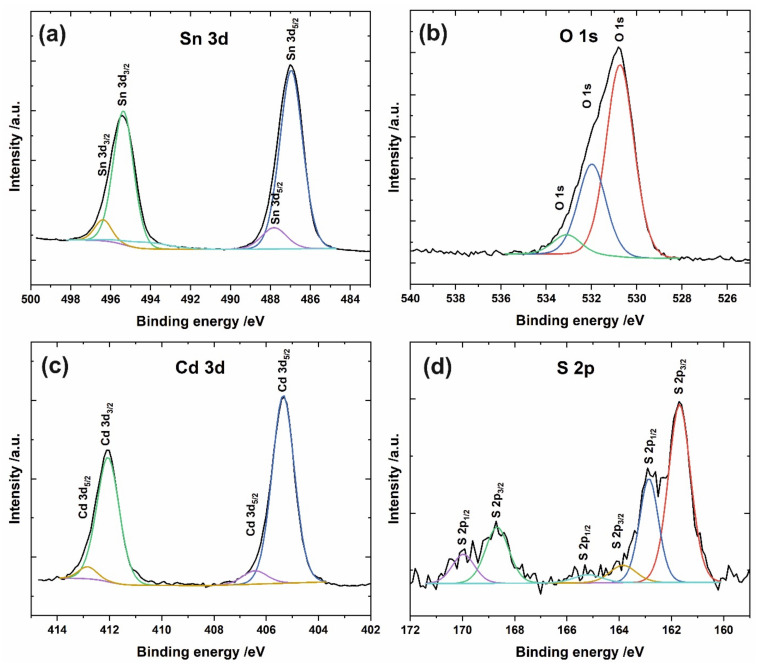
High-resolution XPS spectra of Sn 3d (**a**), O 1s (**b**), Cd 3d (**c**), and S 2p (**d**) recorded for the a/a sample.

The XPS survey spectrum of the a/a sample (see Figure S4 in Supplementary Information) confirmed the presence of Sn, O, Cd, and S on the surface of anodic film after surface modification. The presence of C is the result of typical carbon-containing contaminations adsorbed on the surface during its exposure to the atmosphere. The high-resolution Sn 3d spectrum (Figure 3a) is dominated by two peaks at ca. 487.0 eV and 495.4 eV with a peak separation of 8.4 eV, which can be ascribed to Sn 3d_5/2_ and Sn 3d_3/2_, respectively, and correspond to Sn^4+^-O in SnO_2_ [29,30,31,32]. Slight peaks at 487.8 eV and 496.2 eV may be attributed to some non-stoichiometric tin oxides or organometallic species originating from the anodic oxidation of the Sn foil. Two sharp peaks at 405.4 eV and 412.1 eV in the Cd 3d spectrum (Figure 3c) with typical splitting energy of 6.7 eV confirm the presence of CdS on the sample surface [33,34,35]. This is in agreement with the S 2p spectrum (Figure 3d), with a characteristic doublet of S 2p_3/2_ and S 2p_1/2_ at 161.7 eV and 162.9 eV typically observed for sulfide ions (S^2−^) [34,35]. The pair of small peaks at ca. 168.7 eV and 170.0 eV suggest the possible presence of some species containing an oxidized form of sulfur (e.g., sulfates) [33], while the peaks at around 163.9 eV and 165.2 eV can be attributed to sulfur or the R-SH groups [33]. However, the ratio of Cd to S was found to be close to 1, independently of the sample type. Moreover, no significant differences in the qualitative surface composition between particular samples have been noticed (positions of all aforementioned peaks varied max by 0.1 eV without any noticeable trend). A detailed inspection of the qualitative surface composition reveals that the amount of oxidized forms of sulfur is higher for the samples subjected to the thermal treatment in the air after deposition of CdS. No other differences between all studied samples were observed.

**Figure 4:**
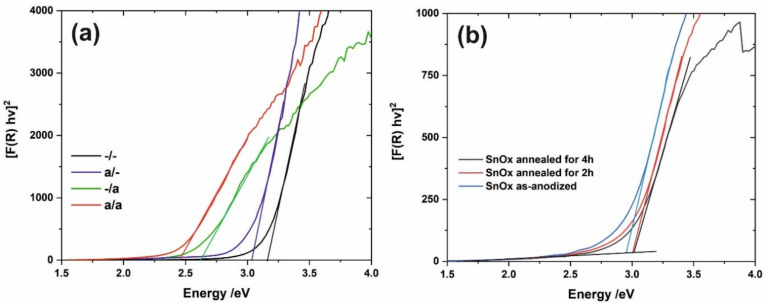
[F(R) hv]^2^ vs. hv plots for all studied nanoporous SnO_x_/CdS (**a**) and unmodified porous SnO_x_ (**b**).

Optical band gap (E_g_) values were estimated from [F(R) hv]^2^ vs. hv plots (Tauc plots) constructed based on UV-Vis DRS spectra according to the previously described procedure [36] (direct nature of E_g_ was assumed). As can be seen in Figure 4, the E_g_ of the samples not subjected to annealing after the SILAR procedure (Figure 4a blue and back lines) are almost the same as those observed for unmodified SnO_x_ (Figure 4b). On the contrary, further annealing of samples modified with CdS results in a noticeable band gap narrowing (Figure 4a—green and red lines) and the narrowest E_g_ of ca. 2.4 eV is observed for the a/a sample. The value is in excellent agreement with this observed typically for pure CdS (2.42 eV), indicating that cadmium sulfide was effectively deposited on the surface of the porous SnO_x_ layer.

**Figure 5:**
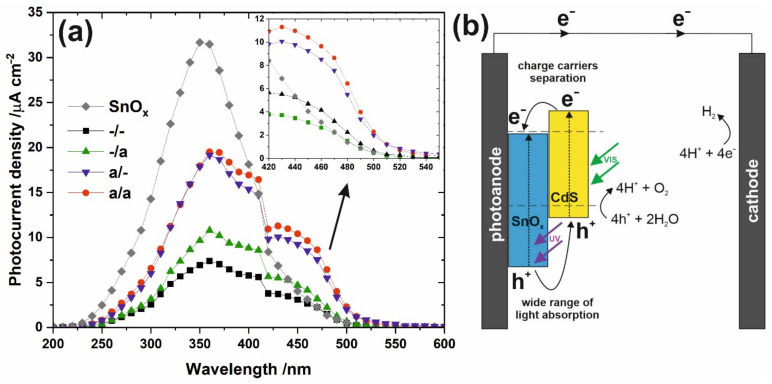
Photocurrent spectra recorded for all studied samples at the potential of 1.0 V vs. SCE in the range between 200 and 550 nm (**a**), together with a schematic representation of the band alignment and charge transfer between SnO_x_ and CdS (**b**).

The photocurrent spectra recorded for all studied nanoporous SnO_x_/CdS photoanodes as well as annealed unmodified SnO_x_ are collected in Figure 5a. Compared to unmodified and annealed SnO_x_, all SnO_x_/CdS samples exhibit much lower photocurrents during illumination with UV light. This may be due to local changes in surface chemistry during further immersion of amorphous SnO_x_ matrix in aqueous solutions of Cd^2+^ and S^2−^ ions (since the worsening of photoresponse in the UV range is much more significant when unannealed samples were modified with CdS). Moreover, the deposition of a thin layer of cadmium sulfide on the tin oxide matrix results in hindered interaction of the wider band gap semiconductor with the light of higher energy. However, at the same time, a dramatic improvement in photoelectrochemical activity in the visible range is observed for the specimens decorated with CdS (see inset in Figure 5a). The photocurrent edge shifts to ca. 510 nm, which perfectly corresponds to the band gap of CdS (ca. 2.4 eV). As expected, thermal treatment of anodic SnO_x_ films before the SILAR procedure seems to be a key factor responsible for the enhanced photoelectrochemical performance due to increased stability of the tin oxide film (as we proved in our previous work [14]) before its further exposure to an aqueous environment. On the contrary, thermal treatment of the sample after deposition of CdS seems to have only a slight positive effect on the photoactivity of the material in the visible range, which can be attributed to the crystallization of cadmium sulfide. At the same time, no effect of post-annealing on the photocurrent values recorded during illumination with UV light was observed, indicating that the tin oxide matrix was unaffected by its further thermal treatment. The enhanced photoelectrochemical properties of SnO_x_/CdS result from both the presence of a narrow band gap semiconductor, which allows the generation of charge carriers during illumination with visible light, as well as favorable positions of the conduction and valence band edges (both are more negative for CdS compared to SnO_x_ as shown in Figure 5b) that facilitates charge carriers separation and reduces the recombination probability.

## 4. Conclusions

In summary, we proved that a thin layer of cadmium sulfide can be successfully deposited on the surface of anodically generated nanoporous tin oxide layers via a simple SILAR method. Moreover, the nanoporous morphology of the as-anodized SnO_x_ film is still maintained after CdS deposition. Such SnO_x_/CdS photoanodes exhibit significantly enhanced photoelectrochemical activity in the visible range due to the lower band gap of CdS (ca. 2.4 eV) and favorable alignment of the band edges. Further investigations are focused on the optimization of the SILAR process (including the number of cycles, solutions composition, the temperature of the process, etc.) to find the optimal thickness of the cadmium sulfide layer as well as possible modification of nanoporous anodic SnO_x_ with other narrow band gap semiconductors.

## Figures and Tables

**Table 1:** Types of SnO_x_/CdS samples used for detailed studies.

Label of the Sample			
-/-	a/-	-/a	a/a
**Annealing after Anodization**			
No	Yes	No	Yes
**Annealing after SILAR Procedure**			
No	No	Yes	Yes

## Data Availability

Not applicable.

## Supplementary Materials

The following supporting information can be downloaded at: https://www.mdpi.com/article/10.3390/ma15113848/s1, Figure S1: FE-SEM image of the as-anodized SnO_x_ layer before deposition of CdS. Figure S2: Higher magnification FE-SEM image of as-anodized SnO_x_ (left) and SnO_x_ modified with CdS after thermal treatment (right); Figure S3: XRD pattern of the a/a sample.; Figure S4: XPS survey spectrum of the a/a sample.
