# Supplementary material for: CdS-Decorated Porous Anodic SnOx Photoanodes with Enhanced Performance under Visible Light
Source: Materials (Basel). 2022 May 27;15(11):3848. doi: 10.3390/ma15113848 (PMC9181453; doi:10.3390/ma15113848)
Supplement: Supplementary file 1 [file materials-15-03848-s001.zip › materials-1706985-supplementary.pdf]

Supplementary Information

CdS-decorated porous anodic SnOx photoanodes with enhanced performance under visible light

Karolina Gawlak[1]*, Dominika Popiołek[1], Marcin Pisarek[2], Grzegorz D. Sulka[1], Leszek Zaraska[1]*

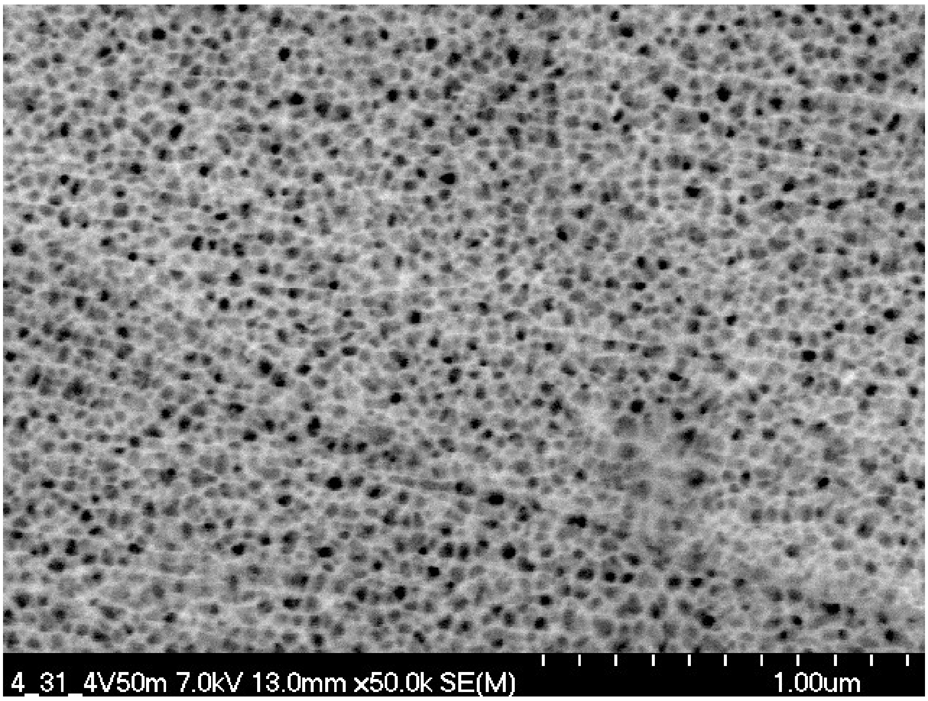

Figure S1. FE-SEM image of the as-anodized SnO_x layer before deposition of CdS.

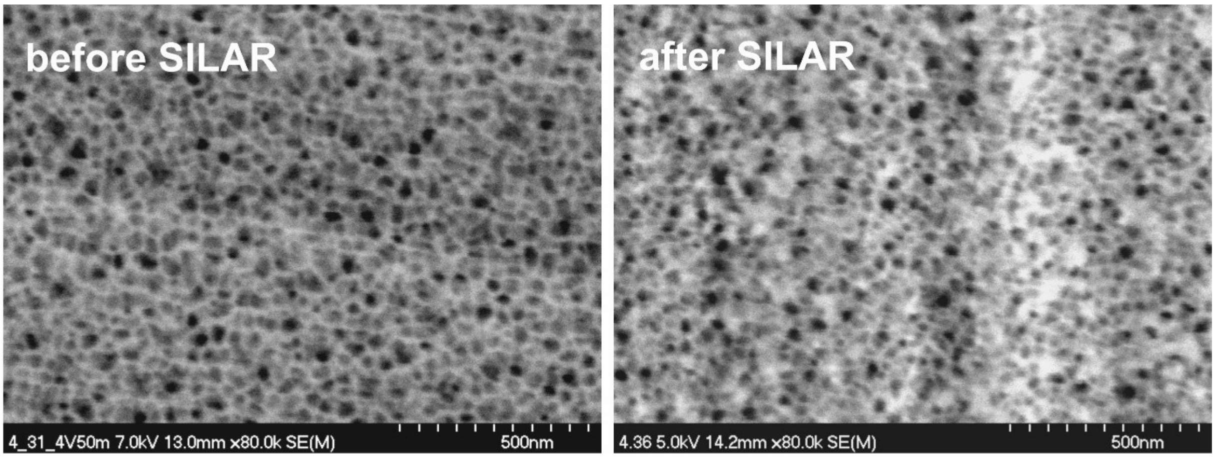

Figure S2. Higher magnification FE-SEM image of as-anodized SnO_x (left) and SnO_x modified with CdS after thermal treatment (right).

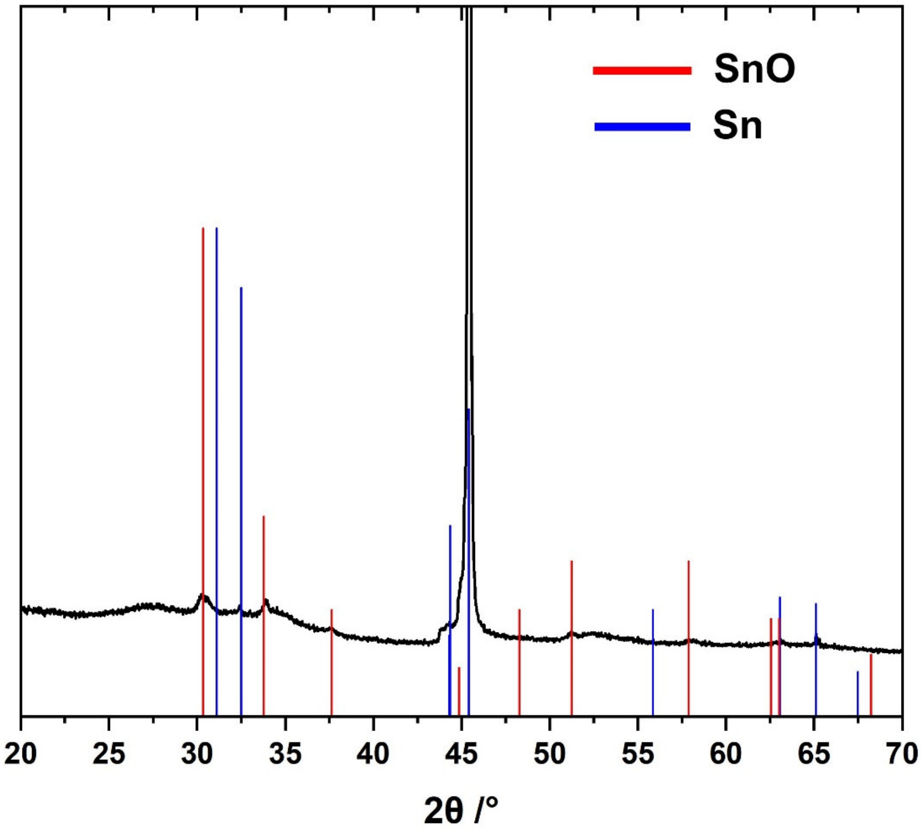

Figure S3. XRD pattern of the a/a sample.

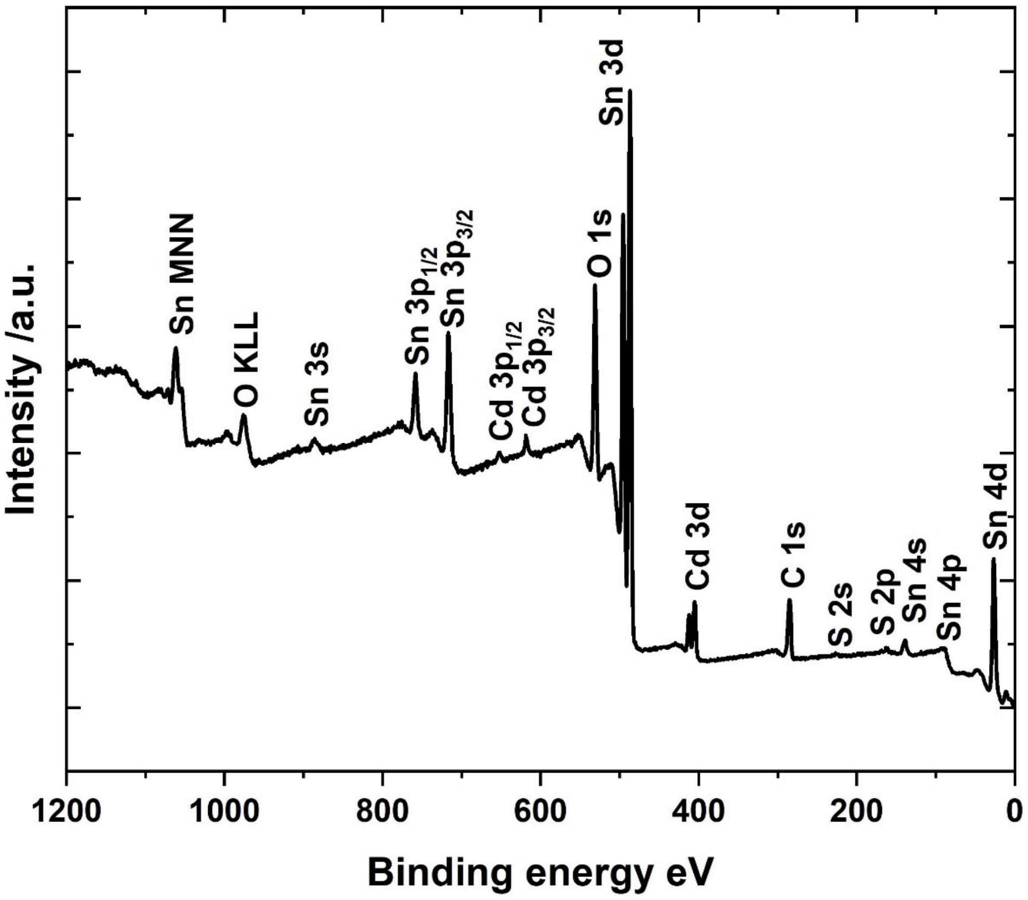

Figure S4. XPS survey spectrum of the a/a sample.
